# Combined use of long-lasting insecticidal nets and *Bacillus thuringiensis israelensis* larviciding, a promising integrated approach against malaria transmission in northern Côte d'Ivoire

**DOI:** 10.1186/s12936-024-04953-8

**Published:** 2024-05-29

**Authors:** Jean-Philippe B. Tia, Emile S. F. Tchicaya, Julien Z. B. Zahouli, Allassane F. Ouattara, Laura Vavassori, Jean-Baptiste Assamoi, Graham Small, Benjamin G. Koudou

**Affiliations:** 1 Université Nangui Abrogoua, Abidjan, Côte d’Ivoire; 2 Centre Suisse de Recherches Scientifiques en Côte d’Ivoire, Abidjan, Côte d’Ivoire; 3 Université Péléforo Gon Coulibaly, Korhogo, Côte d’Ivoire; 4 Centre d’Entomologie Médicale et Vétérinaire, Université Alassane Ouattara, Bouaké, Côte d’Ivoire; 5 University of Basel, Basel, Switzerland; 6 Swiss Tropical and Public Health Institute, Allschwil, Switzerland; 7 Innovative Vector Control Consortium, Pembroke Place, Liverpool, L3 5QA UK

**Keywords:** *Anopheles gambiae*, LLIN, *Bacillus thuringiensis israelensis*, Integrated vector control, Malaria incidence, Côte d’Ivoire

## Abstract

**Background:**

The recent reduction in malaria burden in Côte d’Ivoire is largely attributable to the use of long-lasting insecticidal nets (LLINs). However, this progress is threatened by insecticide resistance and behavioral changes in *Anopheles gambiae *sensu lato (s.l.) populations and residual malaria transmission, and complementary tools are required. Thus, this study aimed to assess the efficacy of the combined use of LLINs and *Bacillus thuringiensis israelensis* (*Bti*), in comparison with LLINs.

**Methods:**

This study was conducted in the health district of Korhogo, northern Côte d'Ivoire, within two study arms (LLIN + *Bti* arm and LLIN-only arm) from March 2019 to February 2020. In the LLIN + *Bti* arm, *Anopheles* larval habitats were treated every fortnight with *Bti* in addition to the use of LLINs. Mosquito larvae and adults were sampled and identified morphologically to genus and species using standard methods. The members of the *An. gambiae* complex were determined using a polymerase chain reaction technique. *Plasmodium* infection in *An. gambiae *s.l. and malaria incidence in local people was also assessed.

**Results:**

Overall, *Anopheles* spp. larval density was lower in the LLIN + *Bti* arm 0.61 [95% CI 0.41–0.81] larva/dip (l/dip) compared with the LLIN-only arm 3.97 [95% CI 3.56–4.38] l/dip (RR = 6.50; 95% CI 5.81–7.29; P < 0.001). The overall biting rate of *An. gambiae* s.l. was 0.59 [95% CI 0.43–0.75] biting/person/night in the LLIN + *Bti* arm against 2.97 [95% CI 2.02–3.93] biting/person/night in LLIN-only arm (P < 0.001). *Anopheles gambiae* s.l. was predominantly identified as *An*. *gambiae *sensu stricto (s.s.) (95.1%, n = 293), followed by *Anopheles coluzzii* (4.9%; n = 15). The human-blood index was 80.5% (n = 389) in study area. EIR was 1.36 infected bites/person/year (ib/p/y) in the LLIN + *Bti* arm against 47.71 ib/p/y in the LLIN-only arm. Malaria incidence dramatically declined from 291.8‰ (n = 765) to 111.4‰ (n = 292) in LLIN + *Bti* arm (P < 0.001).

**Conclusions:**

The combined use of LLINs with *Bti* significantly reduced the incidence of malaria. The LLINs and *Bti* duo could be a promising integrated approach for effective vector control of *An. gambiae* for elimination of malaria.

**Supplementary Information:**

The online version contains supplementary material available at 10.1186/s12936-024-04953-8.

## Background

The burden of malaria remains a significant concern in sub-Saharan Africa, despite the progress made in controlling this disease during the two last decades [1]. The World Health Organization (WHO) has reported recently that there were 249 million cases of malaria worldwide and an estimated 608,000 malaria deaths in 2023 [2]. The WHO African region accounted for 95% of global malaria cases and 96% of deaths attributable to malaria, with the most severely affected being pregnant women and children under 5 years old [2, 3].

Long-lasting insecticidal nets (LLINs) and indoor residual spraying (IRS) have played a key role in reducing the malaria burden in Africa [4]. The scaling-up of these malaria vector control tools led to a 37% drop in the number of malaria cases and a 60% reduction in the mortality rate between 2000 and 2015 [5]. However, the post-2015 trend is worryingly stagnant or even increasing and the level of malaria deaths remains unacceptably high, particularly in sub-Saharan Africa [3]. Several studies have underlined that the emergence and spread of resistance of the major *Anopheles* vectors of malaria to the insecticides used in public health is an obstacle to the future effectiveness of LLINs and IRS [6–8]. In addition, the change in the behaviour of the vectors to biting outdoors and earlier at night is responsible for residual transmission of malaria and is of increasing concern [9, 10]. The limitations of LLINs and IRS in controlling the vectors responsible for residual transmission are a major weakness in current efforts to eliminate malaria [11]. Moreover, the persistence of malaria is attributed to climatic conditions and human activities that contribute to the creation of larval habitats [12].

Larval source management (LSM) is a vector control approach based on targeting mosquito breeding sites with the objective of reducing both the number of breeding sites and the number of mosquito larvae and pupae they contain [13]. LSM has been recommended by several studies as a complementary integrated strategy for malaria vector control [14, 15]. Indeed, the efficacy of LSM offers the dual advantage of targeting malaria vector species that bite both indoors and outdoors [4]. Additionally, vector control through LSM based on a biolarvicide like *Bacillus thuringiensis israelensis* (*Bti*) can increase the panel of malaria control tools. Historically, LSM has played a key role in the successful control of malaria in the USA, Brazil, Egypt, Algeria, Libya, Morocco, Tunisia and Zambia [16–18]. Although, it has played a primary role in integrated pest management in several countries that have eliminated malaria, LSM has not generally been integrated into malaria vector control policy and practices in Africa, being only used in the vector control programmes of a few sub-Saharan countries [14–19]. One reason behind this is that breeding sites are generally considered to be too numerous and difficult to locate, thus making LSM application very expensive to implement [4–11, 13, 14]. As a result, the WHO has recommended for decades that the resources mobilized for malaria vector control should be focused on LLINs and IRS [20, 21]. Only in 2012 did the WHO recommend the integration of LSM, especially *Bti* intervention, as a complement to LLINs and IRS in certain settings in sub-Saharan Africa [20]. Since this WHO recommendation, several pilot studies have been conducted on the feasibility, efficacy and cost of applying biological larvicides in sub-Saharan Africa, and these have demonstrated the efficacy of LSM in reducing *Anopheles* mosquito densities and malaria transmission [22–24].

Côte d'Ivoire is among the top 15 countries in the world where more than three quarters of the global malaria burden occurs [25]. The malaria prevalence in Côte d’Ivoire represents 3.0% of the world malaria burden with an estimated incidence and cases ranging from 300‰ to over 500 ‰ inhabitants [25]. Malaria transmission is perennial in the northern savannah region of the country despite the long dry season from November to May [26]. Malaria transmission in this region is linked to the presence of a high proportion of asymptomatic carriers of *Plasmodium falciparum* [27]. In the region, the most abundant malaria vectors are *Anopheles gambiae *sensu lato (s.l.) populations. Local *An. gambiae* s.l. are predominantly by *Anopheles gambiae *sensu stricto (s.s.), which is strongly resistant to insecticides, thus leading to a high risk of residual malaria transmission [26]. Because of local vector resistance to insecticides, the use of LLINs can may have a limited impact in reducing malaria transmission which, therefore, remains of great concern in this region. Pilot studies based on use of either *Bti* or LLINs have shown effectiveness in lowering mosquito vector densities in the northern region of Côte d’Ivoire. However, no studies have previously assessed the impact of repeated application of *Bti* combined with the use of LLINs on malaria transmission and on malaria incidence in this region. Therefore, the present study was designed to evaluate the impact of the combined use of LLINs and *Bti* on malaria transmission by comparing a LLIN + *Bti* arm with a LLIN-only arm in four villages in the northern region of Côte d’Ivoire. The hypothesis was that a *Bti*-based LSM would add value by further reducing malaria mosquito densities when implemented in addition to LLINs compared the LLINs only being used. Such an integrated approach, targeting both *Anopheles* immatures with *Bti* and adults with LLINs, may be crucial in reducing malaria transmission in an area of high malaria endemicity as the villages of northern Côte d’Ivoire. The outcomes of this study could thus help inform a decision on the possible integration of LSM into the national malaria vector control programmes (NMCPs) of endemic sub-Saharan countries.

## Methods

### Study area

**Fig. 1:**
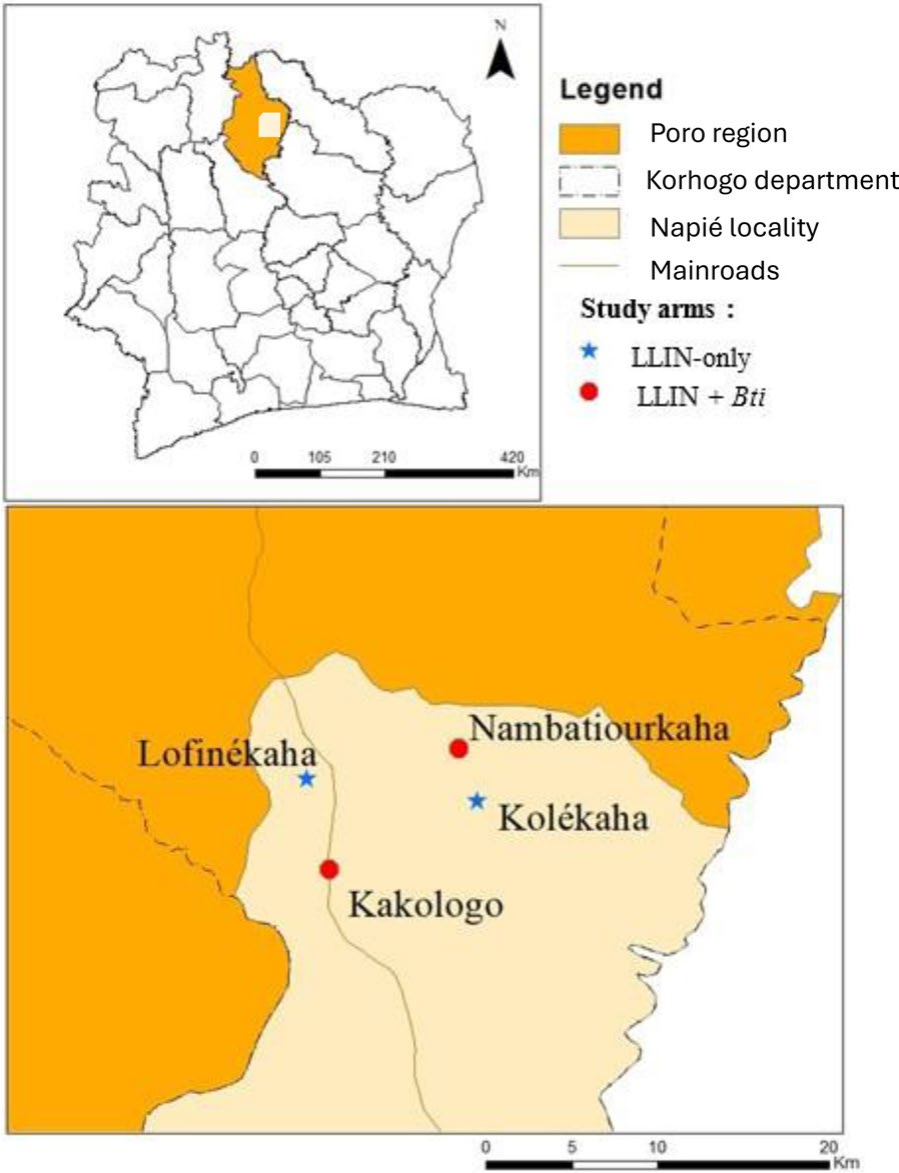
Map of Côte d'Ivoire showing the study areas. (Map source and software: GADM data and ArcMap 10.6.1. *LLIN* Long-lasting insecticidal nets, *Bti* *Bacillus thuringiensis israelensis*

The current study was conducted in four rural villages located in the department of Napiélédougou (also called Napié) in the health district of Korhogo, northern Côte d'Ivoire (Fig. 1). The study villages were Kakologo (9°14′ 2″ N, 5° 35′ 22″ E), Kolékaha (9° 17′ 24″ N, 5° 31′ 00″ E), Lofinékaha (9° 17′ 31″ N, 5° 36′ 24″ E) and Nambatiourkaha (9° 18′ 36″ N, 5° 31′ 22″ E). In 2021, the population of Napiélédougou was estimated at 31,000 inhabitants and this department is composed of 53 villages with two health centres [28]. In Napiélédougou department, malaria is the leading cause of consultations, hospitalization and mortality, and only LLINs are used for *Anopheles* vector control [29]. All four villages in both study arms are served by the same health centre whose records were consulted for clinical malaria cases in this study.

The malaria prevalence was higher 82.0% (2038 cases) in the target population in the Napié health centre (data before *Bti* intervention). In all four villages, householders used only PermaNet® 2.0 LLINs distributed by the NMCP of Côte d’Ivoire in 2017, with a coverage rate of > 80% [25–28, 30]. The villages belonged to the Korhogo region, which is a sentinel site of the NMCP of Côte d'Ivoire and are accessible all year round. Each of the four villages includes at least 100 households, with comparable populations and several cases of malaria were recorded there each year as indicated in the health registers, a working document of the Ministry of Health (MoH) of Côte d’Ivoire). Malaria is mainly caused by *P. falciparum* transmitted to people by *An. gambiae* s.l. with transmission also by *Anopheles funestus* and *Anopheles nili* in the region [28]. The local *An. gambiae* complex consists predominantly of *An. gambiae* s.s*.* with high the frequencies of the *kdr* mutation (frequency range: 90.70–100%) and moderate frequencies of the *ace-1* allele (frequency range: 55.56–95%) [29].

The average annual rainfall and temperature ranged from 1200 to 1400 mm and from 21 to 35 °C, respectively, and the relative humidity (RH) was estimated at 58%. The climate of this study area is Sudanian type, with a 6-month dry season (November to April) and a 6-month rainy season (May to October). The area is experiencing some of the impacts of climate change, such as the disappearance of vegetation cover and a longer dry season marked by the drying up of water bodies (lowlands, rice paddies, ponds, puddles) that can serve as larval habitats for *Anopheles* mosquitoes [26].

The study was conducted in the LLIN + *Bti* arm represented by the villages of Kakologo and Nambatiourkaha and the LLIN-only arm represented by the villages of Kolékaha and Lofinékaha. In all of these villages, people were using only PermaNet® 2.0 LLINs during this study period.

### Study design

The effectiveness of use of LLIN (PermaNet 2.0) in combination with *Bti* against *Anopheles* mosquitoes and malaria transmission were evaluated through a randomized controlled trial (RCT) with two study arms, LLIN + *Bti* arm (treatment arm) and LLIN-only arm (control arm). The LLIN + *Bti* arm was represented by Kakologo and Nambatiourkaha, while Kolékaha and Lofinékaha was designed as the LLIN-only arm. In all four villages, the local populations were using PermaNet® 2.0 LLINs received from the NMCP of Côte d’Ivoire in 2017. The use condition of PermaNet® 2.0 was assumed similar between the villages, because they have received the net in the same way. In the LLIN + *Bti* arm, *Anopheles* mosquito larval habitats were treated with *Bti* every fortnight in addition to the LLINs already being used by the populations. The larval habitats were treated within the villages and within a 2-km radius from the centre of each village as recommended by the WHO and followed by NMCP of Côte d’Ivoire [31]. In contrast, no larviciding treatments with *Bti* were conducted in the LLIN-only arm during the study period.

### Larviciding treatments

The water-dispersible granule formulation of *Bti* (Vectobac WG, 37.4% w/w; lot no. 88–916-PG; 3000 International Toxic Units IUT/mg; Valent BioScience Corp, USA) was used at a dose of 0.5 mg/L. A knapsack sprayer containing 16 L with a fiberglass lance supplied with handle and adjustable nozzle with a flow rate of 52 mL per second (3.1 L/min) was used. For preparing a sprayer containing 10 L of water, the quantity of *Bti* diluted in the suspension was 0.5 mg/L × 10 L = 5 mg. For example, for treating an area with a water volume estimated at 10 L in water with a sprayer containing 10 L, the quantity of *Bti* to dilute was 0.5 mg/L × 20 L = 10 mg. The 10 mg of *Bti* were measured using electronical balance in the field. The suspension was prepared mixing this *Bti* quantity in the graduated pail of 10 L using a spatula. This dose was chosen after a pilot field trial on the efficacy of *Bti* on different larval instars of *Anopheles* spp. and *Culex* spp. under natural conditions in a separate but similar area as the current study area [32]. The amount of suspension of larvicide applied per breeding site and the time duration of application were calculated taking into account the estimated volume of water in the breeding sites [33]. *Bti* was applied using calibrated hand-held sprayers. The sprayers were calibrated and tested in separate exercises and different areas to ensure that the desired amounts of *Bti* were delivered.

To find the best time to treat the larval breeding sites, the team determined a spray window. A spray window is the period during which the applied product provides optimum efficacy: in this study, it lasted from 12 h to 2 weeks, depending on the persistence of *Bti*. The period, from 7 a.m. to 6 p.m., seems necessary to enable larvae present in a breeding site to ingest the *Bti*. Thus, periods of heavy rain were avoided, and when rain meant that spraying had to be stopped treatment was resumed the next day if the weather was favourable. The dates to spray, as well as the exact day and time depended on the weather conditions observed. To calibrate the knapsack sprayer according to the required *Bti* treatment application rate, each technician was trained to visually check and position the sprayer nozzle and maintain the pressure. Calibration was completed by checking that the correct amount of *Bti* treatment was applied evenly per unit area. The larval habitats were treated every fortnight. Larviciding activities were done with the support of four experienced and well-trained technicians. The larviciding activities and participants were supervised by a highly experienced supervisor. The larviciding treatments were started during the dry season, in March 2019. Indeed, the dry season was indicated in a previous study as a the most appropriate time period for the larviciding intervention due to the stability of the breeding sites and their reduced numbers [27]. In anticipation, the control of the larvae during the dry season could prevent the pullulation of the mosquitoes during the rainy season. Two (02) kilograms of *Bti* valued at $99.29 enabled all sites to be covered in the study arm treated. In the LLIN + *Bti* arm, larviciding interventions lasted for a whole year, from March 2019 to February 2020. In total, 22 larviciding treatment events occurred in the LLIN + *Bti* arm.

### Side effect assessment

The potential side effects (e.g., itching, dizziness or nose running) were monitored among the *Bti* biolarvicide sprayers and the occupants of the households involved in the LIN + *Bti* arm through individual interviews.

### Net use rate

Household surveys were conducted among 400 households, made up of 200 households per study arm to assess the percentage of LLINs used in the population. The household surveys were based on quantitative approach using a questionnaire. The rate of use of LLINs was stratified in three age categories: < 5, 5–15 and > 15 years. The questionnaire was addressed and explained in the Sénoufo local language to the heads of the households or other adults over 18 years.

$${\text{Net use rate}} = \frac{{ {\text{Person number having slept on net}}\;{*}\;100}}{{{\text{Total person having slept in hours on the eve of survey}}}}$$

The minimum size of households to be surveyed was calculated using the formula described by Vaughan and Morrow [34].

$${\text{n}} = t^{2} \cdot { }\frac{{p \cdot \left( {1 - p} \right)}}{{e^{2} }}$$

**n** is the sample size, **e** is the margin of error, **t** is the margin coefficient deduced from the confidence rate, **p** is the proportion of the elements of the parent population that have a given property. Each element of this fraction has a conventional value, thus (**t**) = 1.96; **e** = 0.05. The minimum size of households in this condition to survey was 384.

### Entomological surveys

#### Larval collections

Before initiating the current trial, the different types of *Anopheles* mosquito larval habitats were identified, sampled, described, georeferenced and labelled in both LLIN + *Bti* and LLIN arms. The size of the breeding sites was measured using a tape measure. Then, the density of mosquito larvae was assessed every month for 12 months at 30 breeding sites randomly selected per village, totaling 60 breeding sites per study arm. There were 12 larval sampling events corresponding to 22 treatment of *Bti* in each study area arm. The choice of these 30 breeding sites in each in each village was intended to track an equitable number of larval collection points by village and study arm to minimize the biases. A dipper of 60 ml was used to collect the larvae by the dipping method [35]. Use of a small dipper, unlike the standard WHO dipper (350 mL), was necessary due to the specificity of some breeding sites which were very small and shallow. Totals of 5, 10 or 20 dips were taken from the breeding sites of perimeters < 1 m, 1–10 m and > 10 m, respectively. The collected larvae were identified morphologically to genus (e.g., *Anopheles* spp*.*, *Culex* spp. and *Aedes* spp.) directly in the field [36]. Collected larvae were divided into two categories according to development stage: early instar (stage 1 and stage 2) and late instar (stage 3 and stage 4) larvae [37]. Larvae were counted per genus and per stage of development. After counting, mosquito larvae were reintroduced into their specific breeding sites that were refilled to their initial volume with the original water and topped up with rainwater.

The breeding sites were considered positive when at least one larva or pupa of any mosquito genus was present. The larval density was determined by dividing the number of larvae per genus by the number of dips.

#### Adult collection

Adult mosquitoes were collected every two months from ten randomly selected households in each village on two consecutive days per survey. During three consecutive days, a total of 20 households were sampled per study arm throughout the study. Mosquitoes were captured using standard window trap (WT) and pyrethrum spray catch (PSC) methods [38, 39]. First, all houses in each village were numbered. Then, four houses were selected randomly as the adult mosquito collection sites in each village. In each randomly selected house, mosquitoes were collected in the main bedroom. The selected bedrooms had both doors and windows and were occupied by humans on the previous night. The bedrooms were kept closed before to start and during the mosquito collections to prevent mosquitoes from flying out of the room. A WT was installed on each window of each bedroom selected as mosquito sampling point. The next day, mosquitoes that had exited the bedrooms into the WTs were collected early in the morning between 06:00 a.m. and 08:00 a.m. Mosquitoes were collected from the WTs using a mouth aspirator and stored in disposable cups covered with a piece of untreated mosquito net. After WT-based collections, mosquitoes resting inside the same sampled bedrooms were immediately captured using pyrethroid-based PSC. After placing a white sheet on the floor of the bedroom, the doors and windows were closed and the insecticide (active ingredients: 0.25% transfluthrin + 0.20% permethrin) was sprayed. Approximately 10–15 min after spraying, the cover was removed from the sprayed bedroom and mosquitoes that had fallen onto the white sheet were collected with forceps and stored in petri dishes containing water-soaked cotton. The number of people who had stayed overnight in the bedroom sampled was also recorded. The collected mosquitoes were rapidly transferred to the field laboratory for further processing.

### Laboratory procedures

In the laboratory, all the collected mosquitoes were identified morphologically to genus and species [36]. The ovaries of *An. gambiae* s.l. females were dissected in a drop of distilled water on slides, using a binocular dissecting microscope [35]. The parity status was scored to separate parous females from nulliparous females according to morphological aspects of the ovarial tracheoles and determine their parity rate and physiological age [35].

The anthropophilic index was determined by detection of the origin of bloodmeals of freshly blood-fed *An. gambiae* s.l. females among human, livestock (bovine, sheep, goat) and chicken hosts by enzyme-linked immuno-sorbent assay (ELISA) bloodmeal technique [40]. Entomological inoculation rate (EIR) was calculated with human blood fed *An. gambiae* s.l. females estimation [41] In addition, *An. gambiae* s.l. infection with *Plasmodium* malaria parasites was determined by analysing the head and thorax of parous females by using the ELISA-circumsporozoite antigen detection (ELISA-CSP) method [40]. Finally, members of the *An. gambiae* complex were identified by analysing their legs, wings and abdomen by polymerase chain reaction (PCR) technique [34].

The formulas of key entomological outcomes measures used are [38]:

$${\text{Anthropophilic index}} = \frac{{An.\;gambiae\;{\text{s.l. human bloodfed number}}\;{*}\;100}}{{{\text{Total}}\;An.\;gambiae\;{\text{s.l. bloodfed analysed}}}}$$

$${\text{Infection rate}} = \frac{{An.\;gambiae\;{\text{s.l. infected number}}\;{*}\;100}}{{{\text{Total}}\;An.\;gambiae\;{\text{s.l. parous analysed}}}}$$

$${\text{EIR}} = \frac{{An.\;gambiae\;{\text{s.l. human bloodfed estimated}}\;{*}\;{\text{infection rate}}}}{{{\text{Sleeper number by night}}}}$$

EIR is the Entomological infection rate.

### Malaria clinical data collection

Malaria clinical data were obtained from the clinical consultation registers of the Napiélédougou health centre that covered all four villages (i.e., Kakologo, Kolékaha, Lofinékaha and Nambatiourkaha) included in the present study. The review of registers was focused on the records from March 2018 to February 2019 and March 2019 to February 2020. The clinical data from March 2018 to February 2019 have constituted the base data or data before *Bti* intervention and these from March 2019 to February 2020 were the data after *Bti* intervention. The clinical information, age, and village of each patient in both LLIN + *Bti* and LLIN study arms were collected in the health registers. Information such as the village origin, the age, and the diagnosed pathology of each patient was recorded. The case considered in this study as malaria was confirmed by rapid diagnostic tests (RDTs) and/or malaria microscopy test with prescription of artemisinin-based combination therapy (ACT) by a health agents. The malaria cases were subdivided into three age categories (i.e., < 5, 5–15 and > 15 years). The malaria incidence rate per 1000 inhabitants per year was estimated by dividing the rate of the malaria prevalence per 1000 inhabitants by the population size of the village.

The formula of malaria incidence is:

$${\text{Malaria incidence per}} \;1000\;{\text{inhabitants}} = \frac{{{\text{Number of new cases of malaria in year}}\;{*}\;1000}}{{{\text{Total population }}}}$$

### Data analysis

Data collected during the current study were double-entered into a Microsoft Excel database, and then imported into the open source R [42] version 3.6.3 software for the statistical analysis. Package ggplot2 was used to plot the graphs. The generalized linear model with Poisson regression was used to compare larval density and the mean number of bites of mosquitoes per person per night between the study arms. Rate ratio (RR) measure of association was used to compare the average larval density and biting rate of culicids and *An. gambiae* s.l. in between both study arms using the LLIN + *Bti* arm as the baseline. Effect sizes were expressed as odds ratio and 95% confidence interval (95% CI). The rate ratio (RR) of Poisson test was used to compare the proportions and the malaria incidence before and after *Bti* intervention in each study arm. The significance level used was 5%.

### Ethical considerations

The study protocol was approved by the National Ethics Committee for Research of Ministry of Health and public Hygiene in Côte d’Ivoire (N/Ref: 001//MSHP/CNESVS-kp) and the health district and administrative authorities of the region of Korhogo. Signed informed consent was obtained from the household survey participants and the owners and/or residents before collecting mosquito larvae and adults. Household and clinical data was anonymized and kept confidential, with access restricted to the designated investigators only.

## Results

### Effects of *Bti* on mosquito larvae

#### Mosquito larval habitats and composition

A total of 1198 breeding sites were visited. Among these breeding sites inspected in the entire study area 52.5% (n = 629) were in the LLIN + *Bti* arm and 47.5% (n = 569) in the LLIN-only arm (RR = 1.10 [95% CI 0.98–1.24], P = 0.088). Overall, the local larval habitats were categorized into 12 types, with rice paddies accounting for the highest proportion of larval habitats (24.5%, n = 294), followed by reflows of showers (21.0%, n = 252), earthenware vessels (8.3%, n = 99), river edges (8.2%, n = 100), pools (7.2%, n = 86), puddles (7.0%, n = 84), village pumps (6.8%, n = 81), hoof prints (4.8%, n = 58), swamps (4.0%, n = 48), jars (5.2%, n = 62), ponds (1.9%, n = 23) and watering wells (0.9%, n = 11).

Overall, a total 47,274 mosquito larvae were collected in the whole study area, with substantially lower proportion of 14.4% (n = 6796) in LLIN + *Bti* arm compared with 85.6% (n = 40,478) in LLIN-only arm (RR = 5.96 [95% CI 5.80–6.11], P ≤ 0.001). These larvae were composed of three mosquito genera, dominated by *Anopheles* spp. (48.7%, n = 23,041), followed by *Culex* spp. (35.0%, n = 16,562) and *Aedes* spp. (4.9%, n = 2340). Pupae represented 11.3% of immature culicids (n = 5344).

#### *Anopheles* spp. larval density

The overall mean larval density of *Anopheles* spp. was 0.61 [95% CI 0.41–0.81] larvae per dipper (l/dip) in the LLIN + *Bti* arm and 3.97 [95% CI 3.56–4.38] l/dip in LLIN-only arm during this trial (Additional file 1: Fig. S1). The mean density of *Anopheles* spp. was 6.5-fold high in LLIN-only arm than in LLIN + *Bti* arm (RR = 6.49; 95% CI 5.80–7.27; P < 0.001). During the treatment period, no one *Anopheles* spp. larvae was collected in the LLIN + *Bti* arm from January corresponding to twentieth *Bti* treatment. The significant reduction in larval density in LLIN + *Bti* arm was observed in both the early and late instars.

##### Early instar

**Fig. 2:**
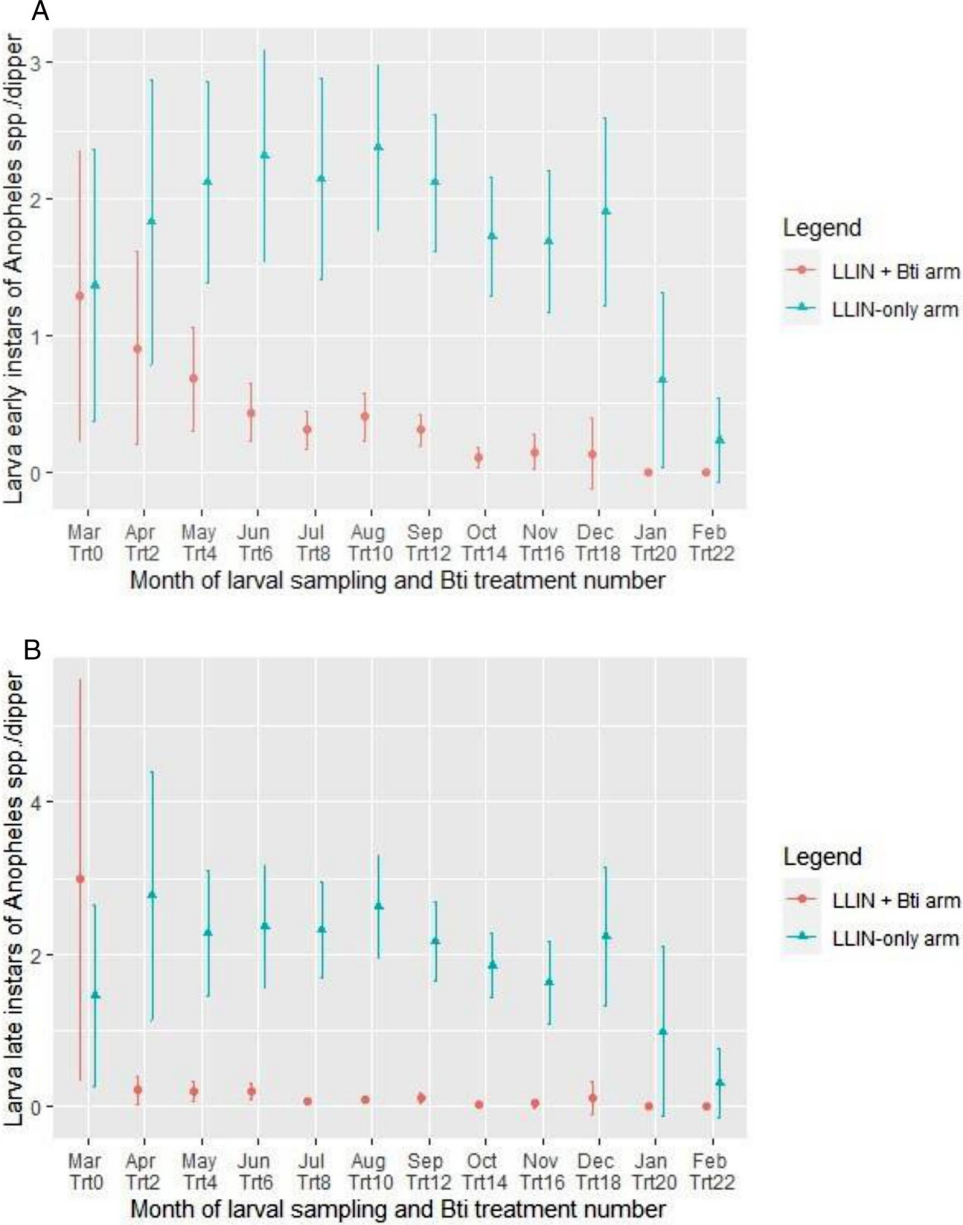
Variation in the average density of larvae of *Anopheles* spp. of early instar (**A**) and of late instar (**B**) in the study arms, in Napié area in northern Côte d’Ivoire, from March 2019 to February 2020. LLIN: long-lasting insecticidal nets; *Bti*: *Bacillus thuringiensis israelensis;* Trt: treatment

Before the *Bti* treatment starting (in March), the mean density of early instars of *Anopheles* was estimated at 1.28 [95% CI 0.22–2.35] l/dip in the LLIN + *Bti* arm and 1.37 [95% CI 0.36–2.36] l/dip in the LLIN-only arms (Fig. 2A). After the *Bti* treatment was applied, the mean density of early instar of *Anopheles* generally decreased gradually from to 0.90 [95% CI 0.19–1.61] to 0.10 [95% CI − 0.03–0.18] l/dip in the LLIN + *Bti* arm. In the LLIN + *Bti* arm, the density of *Anopheles* larvae in the early instars has remained low. In the LLIN-only arm, a fluctuation of *Anopheles* spp. Larvae in the early instars was observed, with a mean density from 0.23 [95% CI 0.07–0.54] l/dip to 2.37 [95% CI 1.77–2.98] l/dip. In general, the mean density early instar *Anopheles* larvae of 1.90 [95% CI 1.70–2.10] l/dip was statistically higher in the LLIN-only arm compared to 0.38 [95% CI 0.28–0.47]) l/dip in the LLIN + *Bti* arm (RR = 5.04; 95% CI 4.36–5.85; P < 0.001).

##### Late instar

In the LLIN + *Bti* arm, the mean density of late instar larvae of *Anopheles* spp. before *Bti* treatment was 2.98 [95% CI 0.26–5.60] l/dip whilst the density in the LLIN-only arm was 1.46 [95% CI 0.26–2.65] l/d. Following *Bti* applications, the density of late instar *Anopheles* larvae in the LLIN + *Bti* arm dropped from 0.22 [95% CI 0.04–0.40] to 0.03 [95% CI 0.00–0.06] l/dip (Fig. 2B). In the LLIN-only arm, the density of late *Anopheles* larvae increased from 0.35 [95% CI − 0.15–0.76] to 2.77 [95% CI 1.13–4.40] l/dip with some variation of larval density according to the date of sampling. The mean density late instar *Anopheles* larvae was 2.07[95% CI 1.84–2.29] l/dip obtained in the LLIN-only arm, ninefold higher than the 0.23 [95% CI 0.11–0.36] l/dip in LLIN + *Bti* arm (RR = 8.80; 95% CI 7.40–10.57; P < 0.001).

#### *Culex* spp. larval density

The mean density of *Culex* spp. was 0.33 [95% CI 0.21–0.45] l/dip in the LLIN + *Bti* arm and 2.67 [95% CI 2.23–3.10] l/dip in the LLIN-only arm (Additional file 2: Fig. S2). The mean density of *Culex* spp. was significantly higher in the LLIN-only arm than in the LLIN + *Bti* arm (RR = 8.00; 95% CI 6.90–9.34; P < 0.001).

##### Early instar

**Fig. 3:**
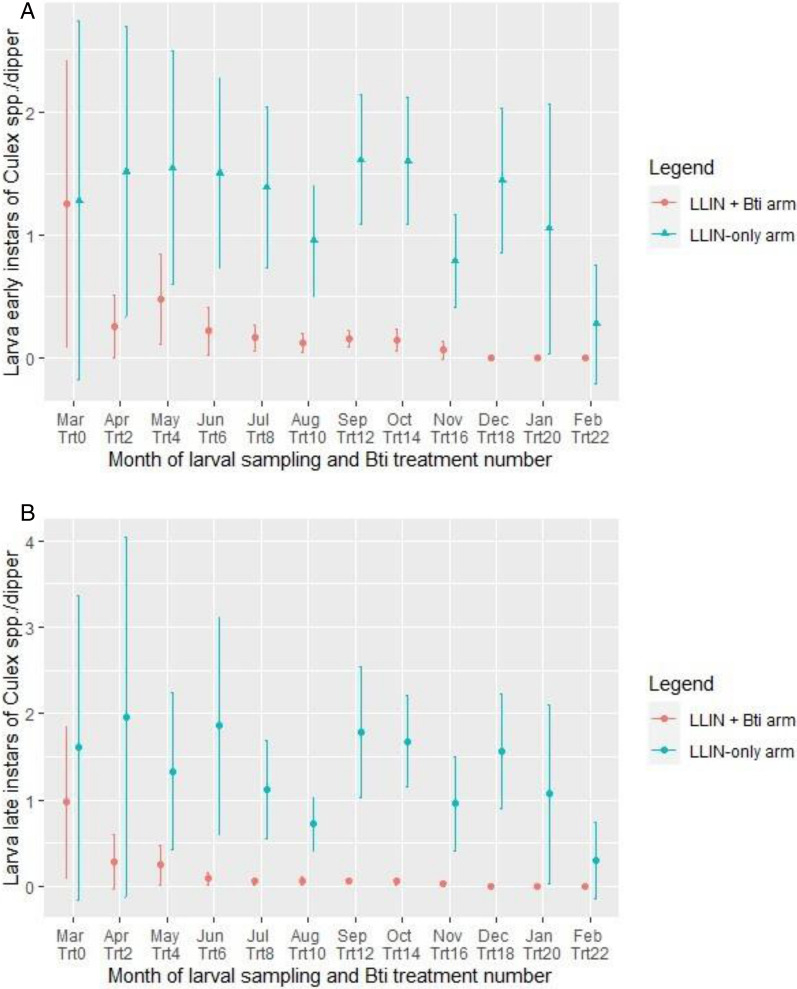
Variation in the average density of larvae of *Culex* spp*.* of early instar (**A**) and early instar (**B**) in the study arms, in Napié area in northern Côte d’Ivoire from March 2019 to February 2020. *LLIN* long-lasting insecticidal nets, *Bti* *Bacillus thuringiensis israelensis*, *Trt* treatment

The mean density of early instar *Culex* spp. before the *Bti* treatment starting was 1.26 [95% CI 0.10–2.42] l/dip in the LLIN + *Bti* arm and 1.28 [95% CI 0.37–2.36] in the LLIN-only arm (Fig. 3A). After *Bti* treatment application, the density of early instar *Culex* larvae reduced, varying between 0.07 [95% CI − 0.001–0.] and 0.25 [95% CI 0.006–0.51] l/dip. From the month of December, no *Culex* larvae were collected from larval habitats treated with *Bti*. In the LLIN + *Bti* arm, the density of early instar *Culex* larvae was reduced to 0.21[95% CI 0.14–0.28] l/dip but in the LLIN-only arm it was higher at 1.30 [95% CI 1.10–1.50] l/d. The density of early instar *Culex* larvae was 6 time higher in the LLIN only arm than in the LLIN + *Bti* arm (RR = 6.17; 95% CI 5.11–7.52; P < 0.001).

##### Late instar

Before the *Bti* treatment, the mean density of late instar *Culex* larvae was 0.97 [95% CI 0.09–1.85] and 1.60 [95% CI − 0.16–3.37] l/dip in LLIN + *Bti* and LLIN arms, respectively (Fig. 3B). Following the initiation of *Bti* treatments, the mean density of late instar *Culex* spp. in the LLIN + *Bti* arm decreased progressively and became lower than in LLIN-only arm where densities remained very high. The mean density of late instar *Culex* larvae was 0.12 [95% CI 0.07–0.15] l/dip in LLIN + *Bti* arm and 1.36 [95% CI 1.11–1.61] l/dip in the LLIN-only arm. The mean density of late instar *Culex* larvae was significatively higher in the LLIN-only arm than in the LLIN + *Bti* arm (RR = 11.19; 95% CI 8.83–14.43; P < 0.001).

### Effects of *Bti* on pupa density of culicid fauna

**Fig. 4:**
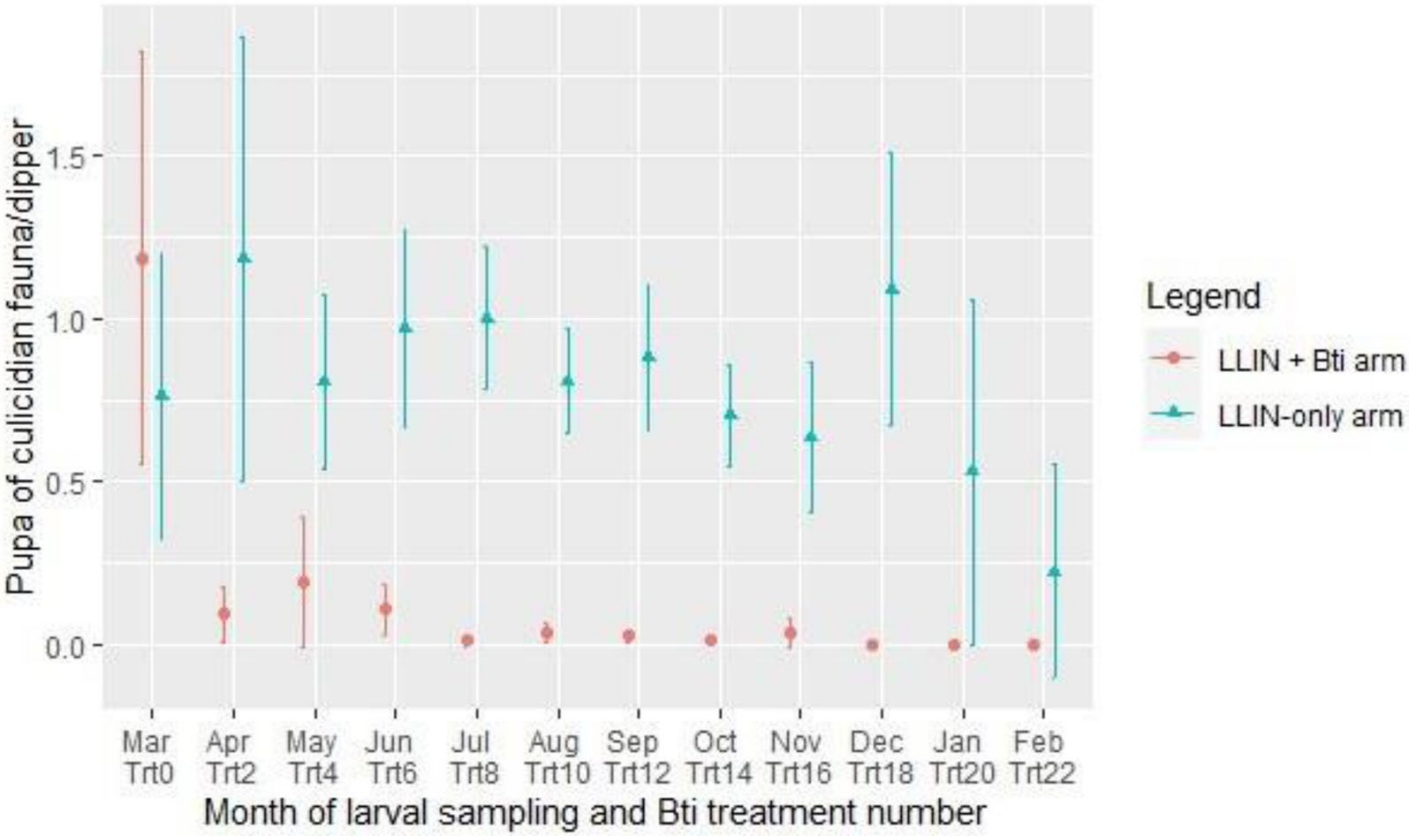
Variation in the average density of pupae*.* in the study arms, in Napié area in northern Côte d’Ivoire from March 2019 to February 2020. *LLIN* long-lasting insecticidal nets, *Bti* *Bacillus thuringiensis israelensis*, *Trt* treatment

Before the *Bti* treatments started, mean density of pupae per dipper was 0.59 [95% CI 0.24–0.94] in the LLIN + *Bti* arm and 0.38 [95% CI 0.13–0.63] in the LLIN-only arm (Fig. 4). The overall density of the pupae was 0.10 [95% CI 0.06–0.14] in the LLIN + *Bti* arm against 0.84 [95% CI 0.75–0.92] in the LLIN-only arm. The *Bti* treatment reduced significatively pupal mean density in the LLIN + *Bti* arm compared to the LLIN-only arm (OR = 8.30; 95% CI 6.37–11.02; P < 0.001). In the LLIN + *Bti* arm, no pupae were collected after the month of November.

### Effects of *Bti* on adult mosquitoes and malaria transmission

#### Species composition

A total of 3456 adult mosquitoes were collected in the study area. Mosquitoes belonged to five genera (*Anopheles*, *Culex, Mansonia*, *Aedes* and *Eretmapodites*) and 17 species (Table 1). Among the malaria vectors, *An. gambiae* s.l. was the most abundant species, with a proportion of 74.9% (n = 2587), followed by *An. funestus* (2.5%, n = 86), and *An. nili* (0.7%, n = 24). The abundance of *An. gambiae* s.l. was lower in the LLIN + *Bti* arm (10.9%, n = 375) than in the LLIN-only arm (64%, n = 2212). No *An. nili* individuals were collected in the LLIN-only arm. However, *An. gambiae* and *An. funestus* were present in both the LLIN + *Bti* and LLIN-only arms.

**Table 1:** Abundance of adult mosquitoes collected during entomological surveys carried out in households from March 2019 to February 2020, in Napié area in northern Côte d’Ivoire

Mosquito species	Study arms					
	LLIN + Bti		LLIN-only		Total	
	n	%	n	%	n	%
*An. gambiae* s.l	375	10.9	2212	64	2587	74.9
*An. funestus*	33	1	53	1.5	86	2.5
*An. nili*	24	0.7	0	0	24	0.7
*An. pharoensis*	4	0.1	26	0.8	30	0.9
*An. ziemanni*	2	0.1	3	0.1	5	0.1
*Ae. aegypti*	73	2.1	114	3.3	187	5.4
*Ae. opok*	3	0.1	8	0.2	11	0.3
*Ae. papalis*	0	0	1	0.0	1	0
*Ae. vittatus*	9	0.3	7	0.2	16	0.5
*Cx. annulioris*	28	0.8	16	0.5	44	1.3
*Cx. cinerus*	2	0.1	14	0.4	16	0.5
*Cx. nebulosus*	157	4.5	201	5.8	358	10.4
*Cx. quinquefasciatus*	14	0.4	34	1	48	1.4
*Cx. tigripes*	6	0.2	7	0.2	13	0.4
*Er. chrysogaster*	4	0.1	1	0.0	5	0.1
*Ma. africana*	0	0	13	0.4	13	0.4
*Ma. uniformis*	7	0.2	5	0.1	12	0.3
Total	741	21.4	2715	78.6	3456	100

n, Number; %, Percentage; LLIN, long-lasting insecticidal-treated net; *Bti*, *Bacillus thuringiensis var. israelensis*;* An*,* Anopheles*; s.l., sensu lato;* Ae*, *Aedes*,* Cx*,* Culex*;* Er*,* Eretmapodites*;* Ma*,* Mansonia*

#### Culicid nuisance

**Fig. 5:**
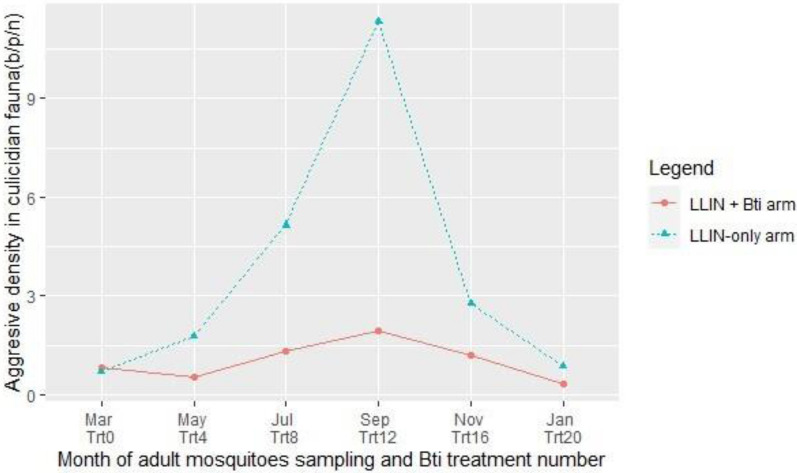
Biting rate in culicidian fauna in study arms in Napié area in northern Côte d’Ivoire, from March 2019 to February 2020. *LLIN* long-lasting insecticidal nets, *Bti* *Bacillus thuringiensis israelensis*, *Trt* treatment, *b/p/n* biting/person/night

In study starting before (March) *Bti* application in the breeding sites, the overall mean biting of culicids per person by night (b/p/n) was estimated at 0.83 [95% CI 0.50–1.17] in LLIN + *Bti* arm against 0.72 [95% CI 0.41–1.02] in LLIN-only arm (Fig. 5). In LLIN + *Bti* arm, culicid nuisance diminished and remained low, although there was a peak of 1.95 [95% CI 1.35–2.54] b/p/n in September after twelfth application of *Bti*. However, mosquito mean biting rate increased progressively before reaching a peak of 11.33 [95% CI 7.15–15.50] b/p/n in September in LLIN-only arm. In LLIN + *Bti*, the overall biting rate of mosquitoes was significatively lower compared to LLIN-only arm at any time point during the trial (RR = 3.66; 95% CI 3.01–4.49; P < 0.001).

#### Malaria transmission

**Fig. 6:**
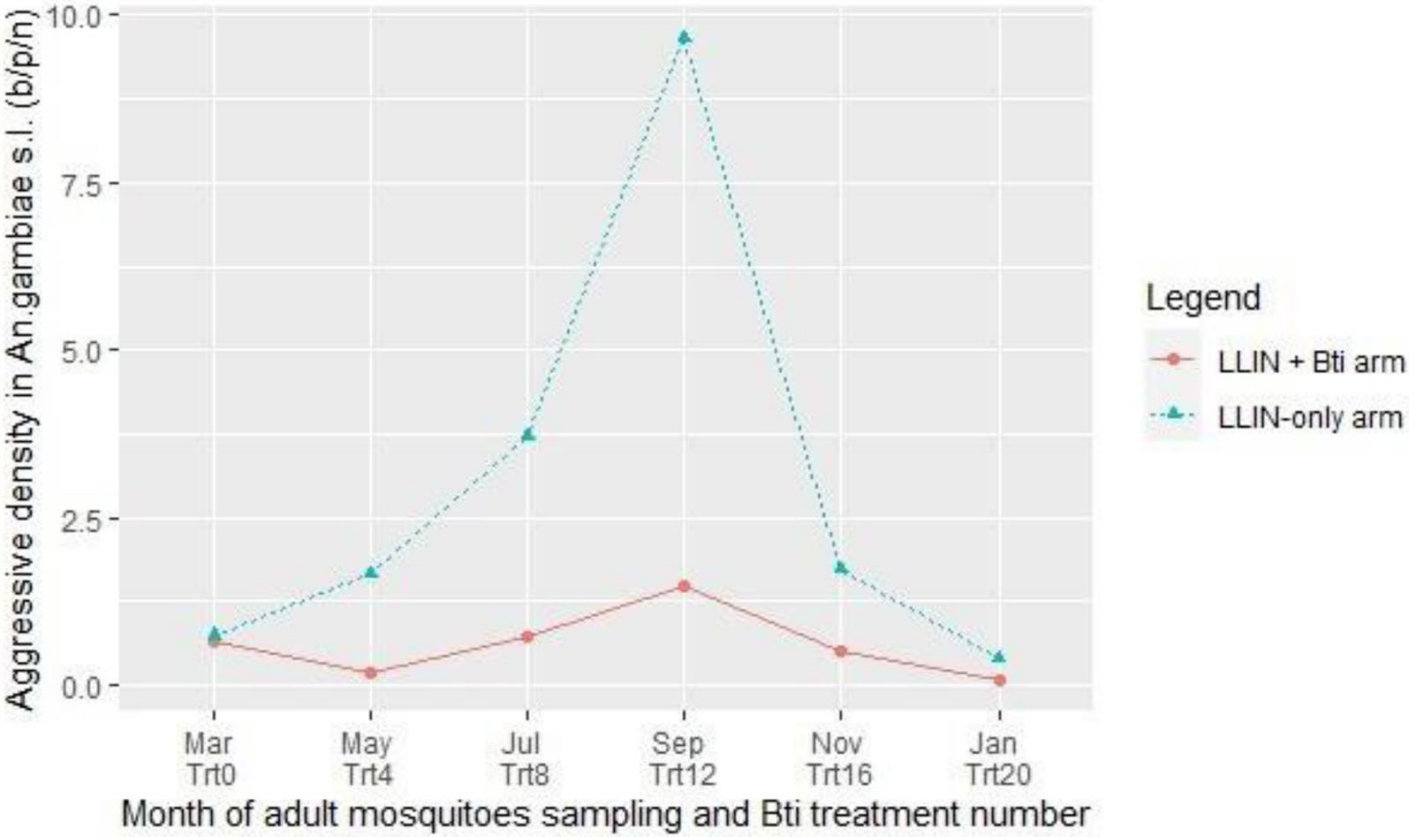
Biting rate in *An. gambiae* s.l., in study arms in Napié area in northern Côte d’Ivoire, from March 2019 to February 2020. *LLIN* long-lasting insecticide-treated nets, *Bti* *Bacillus thuringiensis israelensis*, *Trt* treatment, *b/p/n* biting/person/night

*Anopheles gambiae* s.l. was the most abundant malaria vector in the study area. The biting rate of *An. gambiae* females was 0.64 [95% CI 0.27–1.00] b/p/n in the LLIN + *Bti* arm and 0.74 [95% CI 0.30–1.17] in the LLIN-only arm at the beginning of study (Fig. 6). During the *Bti* intervention, the highest biting rate activities were observed in September month corresponding to twelfth *Bti* treatment, with a peak of 1.46 [95% CI 0.87–2.05] b/p/n in LLIN + *Bti* arm and 9.65 [95% CI 5.23–14.07] in LLIN-only arm. The overall biting rate of *An. gambiae* in LLIN + *Bti* arm (0.59 [95% CI 0.43–0.75] b/p/n) was significantly lower than in LLIN-only arm (2.97 [95% CI 2.02–3.93] b/p/n) (RR = 3.66; 95% CI 3.01–4.49; P < 0.001).

A total of 646 *An. gambiae* were dissected. Overall, the percentage of local *An. gambiae* parous was generally > 70% throughout the study except in July in the LLIN-only arms (Additional file 3: Fig. S3). However, the mean parity rate was 74.5% (n = 481) in study area. In LLIN + *Bti* arm, the parity rate has remained high and superior at 80% to exception September month where it reduced to 77.5%. Whereas a fluctuation was observed in parity rate mean in LLIN-only arm, with lowest parity rate mean estimated 64.5%.

Out of the 389 *An. gambiae* individual bloodmeals examined, 80.5% (n = 313) originated from humans, 6.2% (n = 24) of the females had a mixed blood meal (human and livestock) 5.1% (n = 20) had a blood meal from livestock (bovine, sheep, and goat) blood and 8.2% (n = 32) of specimens analysed were negative for a blood meal. In the LLIN + *Bti* arm, the females having a human blood meal was25.7% (n = 100) against 54.8% (n = 213) in LLIN-only arm (Additional file 5: Table S5).

A total of 308 *An. gambiae* were tested for determining species complex members and infection with *P. falciparum* (Additional file 4: Table S4). In the study area, two “sibling species” were coexisting, namely *An. gambiae* s.s*.* (95.1%, n = 293) and *An. coluzzii* (4.9%, n = 15)*. Anopheles gambiae* s.s. was significatively less predominant (28.9%, n = 89) in the LLIN + *Bti* arm than in the LLIN-only arm (66.2%, n = 204) (RR = 2.29 [95% CI 1.78–2.97], P < 0.001). *Anopheles coluzzii* was found in equal proportions in the LLIN + *Bti* arm (3.6%, n = 11) and in the LLIN-only arm (1.3%, n = 4) (RR = 2.75 [95% CI 0.81–11.84], P = 0.118). The *P. falciparum* infection rate within *An. gambiae* s.l. was 11.4% (n = 35). The *P. falciparum* infection rate in *An. gambiae* was significantly lower in the LLIN + *Bti* arm (2.9%, n = 9) than in the LLIN-only arm (8.4%, n = 26) (RR = 2.89 [95% CI 1.31–7.01], P = 0.006). *Anopheles gambiae* s.s. was the most infected with *Plasmodium* at 94.3% (n = 32) compared to *An. coluzzii* at only 5.7% (n = 5) (RR = 6.4 [95% CI 2.47–21.04], P < 0.001).

### Net use rate

A total of 400 households with a population of 2435 inhabitants were surveyed. The average density was 6.1 persons per household. The LLINs ownership rate in households was 85% (n = 340) compared with 15% (n = 60) which did not own an LLIN (RR = 5.67 [95% CI 4.29–7.59], P < 0.001) (Additional file 5: Table S5**)**. The LLIN use rate was 40.7% (n = 990) in LLIN + *Bti* arm against 36.2% (n = 882) in LLIN-only arm (RR = 1.12 [95% CI 1.02–1.23], P = 0.013). The mean overall net use rate was 38.4% (n = 1842) in the study area. The children under the age of five used the net in approximately equal proportions in the both study arms with the net use rate 41.2% (n = 195) in LLIN + *Bti* arm and 43.2% (n = 186) in LLIN-only arm (RR = 1.05 [95% CI 0.85–1.29], P = 0.682). The net use rates were not different in children between the ages of 5 to 15 years in LLIN + *Bti* arm 36.3% (n = 250) and in LLIN-only arm 36.9% (n = 250) (RR = 1.02 [95% CI 1.02–1.23], P = 0.894). However, the people over 15 years used the nets less in the LLIN + *Bti* arm 42.7% (n = 554) than in the LLIN-only arm 33.4% (n = 439) (RR = 1.26 [95% CI 1.11–1.43], P < 0.001).

### Impact of Bti intervention on clinical malaria incidence

A total of 2484 clinical cases were recorded in the Napié health centre for the period from March 2018 to February 2020. Clinical malaria prevalence in the general population represented 82.0% (n = 2038) of all clinical cases of pathologies. Local malaria incidence per year was 479.8‰ and 297.5‰ before and after *Bti* treatment in the present study area (Table 2).

**Table 2:** Impact of *Bti* intervention on malaria incidence from March 2018 to February 2020 in the target population in Napié area

Study arm	Population	Age groups	Before *Bti* treatment		After *Bti* treatment		Poisson test	
			Malaria cases	MI (‰)	Malaria cases	MI (‰)	RR [95% CI]	P-value
LLIN + *Bti* arm	2622	< 5 years	359	136.9a	131	50.0b	0.58 [0.47–0.72]	< 0.001
		5–15 years	229	87.3a	87	33.2b	0.61 [0.47–0.78]	< 0.001
		> 15 years	177	67.5a	74	28.2b	0.67 [0.51–0.88]	< 0.001
		n	765	291.8a	292	111.4b	0.38 [0.33–0.44]	< 0.001
LLIN-only arm		< 5 years	231	88.1a	253	96.5a	1.11 [0.92–1.32]	0.286
		5–15 years	145	55.3a	127	48.4a	0.89 [0.69–1.13]	0.344
		> 15 years	117	44.6a	108	41.2a	0.93 [0.71–1.22]	0.648
		n	493	188.0a	488	186.1a	0.99 [0.87–1.12]	0.898

LLIN, long-lasting insecticidal nets; *Bti*, *Bacillus thuringiensis var. israelensis*; MI, Malaria incidence; ‰, per 1000 inhabitants; %, percentage n: Total malaria cases in study area; RR, Rate ration; 95%CI, Confidence interval at 95%

Same letter denoted no difference between the malaria incidence before and after *Bti* intervention P > 0.05

Different letter denoted Difference between malaria incidence before and after Bti intervention P < 0.05

In the LLIN + *Bti* arm, malaria incidence was reduced significantly from 291.8‰ (n = 765) before the *Bti* intervention to 111.4‰ (n = 292 cases) after the *Bti* intervention, (RR =  = 0.38 [0.33–0.44]; P < 0.001]. However, malaria incidence per year remained stable and higher in the LLIN-only arm with 4188.0‰ before the initiation of the *Bti* intervention and 186.1‰ (n = 488 cases) after this intervention, in all age groups (RR 0.99 [0.87–1.12]; P = 0.898]).

### Perceived side effects

There were no negative side effects reported among the sprayers and the people of the of the villages treated with *Bti* biolarvicide either during or after applications.

## Discussion

Recent progress in the reduction of malaria burden, which has largely been attributable to deployment of LLINs and IRS, is now threatened by *Anopheles* vector resistance to insecticides and behavioural changes, residual transmission, and low use of LLINs [2]. Thus, the current study aimed at producing evidence on the possible benefits of combining LLINs and *Bti*-based larviciding in reducing local *An. gambiae* s.l*.* densities and malaria transmission and clinical incidence in comparison with LLINs alone in the rural savannah villages of northern Côte d’Ivoire. The results of the current study showed that repeated applications of *Bti* biolarvicide to *Anopheles* mosquito larval habitats in the presence of LLINs reduced significantly the larval and adult densities of *An. gambiae* and the transmission and number of clinical cases of malaria in the LLIN + *Bti* arm compared with LLIN-only arm. These outcomes demonstrated the added value of integrating biolarvicide-based LSM programmes into the mass distribution of LLINs to combat residual malaria transmission in rural sub-Saharan African settings.

The current field study was conducted in high malaria transmission areas of northern Côte d’Ivoire where *Anopheles* mosquitoes and other several key arboviral and filarial disease vectors (*Cx. quinquefasciatus* and *Ae. aegypti*) co-occur. Indeed, while only *Anopheles* mosquitoes were targeted in this study, the collected Culicidae fauna comprised 17 species, with a predominance of the malaria primary vector *An. gambiae* and presence of secondary vectors *An. funestus* and *An. nili*. The high species diversity and high abundance of mosquitoes in the study area could be attributed the presence of various types of larval habitats and to a limited impact of LLINs distributed through the national campaigns of mass LLIN distribution led by the NMCP of Côte d’Ivoire. The LLINs use rate among the target population was 76.9% with a net use rate very low in each study arm. This low rate of net use observed could be explained by the fact that this study was carried out during the second year after the distribution. The wear of the fabrics could lead to the abandonment of certain nets. It is also likely that part of the population would not like to use the LLINs because of the heat or that the LLINs are used for other purposes. Although access to LLINs is made possible and facilitated thanks to the joint efforts of governments and partners, the sustainable mass use of this control tool remains a challenge. For a better protection of the population against the infective bites of the vectors, the LSM in complement to LLINs should be an option of choice in the decision-makers.

In this study, local mosquito immatures (larvae and pupae) were collected from aquatic habitats, including rice paddies, reflows of showers, earthenware vessels, river edges, pools, village pumps, puddles, animal hoof prints, swamps, jars, ponds, and watering wells. The predominance of the highly anthropophilic *An. gambiae* s.l., composed mainly of *An. gambiae* s.s. (95%) co-existing with *An. coluzzii*, could be explained by the existence of rice fields and puddles but also frequent human blood-feeding opportunities as only < 40% of local households were using LLINs. Such a low use rate of LLINs (< 80% LLIN universal coverage recommended by WHO) is common in rural sub-Saharan villages and could be due to limited ownership and access to LLINs, and not using nets due to the heat. The abundance of human-biting and parous *An. gambiae* populations combined with a low use of LLIN may explain the high transmission of *P. falciparum* and high numbers of clinical malaria cases found in the present study areas. Therefore, these areas represented appropriate settings for testing the efficacy of adding *Bti* biolarvicide to LLINs to control *An. gambiae* and malaria transmission in rural villages of sub-Saharan Africa.

In the present study, after the *Bti* larviciding of larval habitats was initiated in the LLIN + *Bti* arm, the density of *Anopheles* spp. and *Culex* spp. immatures (i.e., early instar larvae, late instar larvae and pupae) gradually decreased in the LLIN + *Bti*-intervention arm, leading to significantly lower mosquito larval and adult densities in the LLIN + *Bti* arm compared to the LLIN-only arm. Similar observations have been reported from previous studies that showed the effectiveness of a *Bti* larviciding intervention on mosquito immatures in Kenya [22] and Burkina Faso [23], and other African settings [23]. Early studies have demonstrated the effectiveness of *Bti* on *An. gambiae* and *Cx. quinquefasciatus* larvae in rice growing areas of Côte d'Ivoire [31] and Tanzania [43]. *Bti* is a biological or a naturally occurring bacterium found in soils and containing effective against the immature forms of insects, including the mosquito larvae. The *Bti* is ingested by larvae in the form of crystals released from the spores which are transformed into protoxin molecules. These molecules, under the action of intestinal protease in the target organism, release cytolytic (Cyt1Aa) protoxin which cross the peripheral membrane for fixing on over specific receiver situated on the intestinal epithelium surface. The accumulation of Cyt1Aa in the epithelium cell entrains firstly the alimentary canal paralysis and then the intestinal cell lysis of the mosquito larvae [44]. *Bti* has been shown to have no toxicity to people and is approved for use for pest control in organic farming operations. There is no documented mosquito larval resistance to *Bti* as a biolarvicide to date [44]. *Bti* has been found to control effectively pyrethroid resistant *Anopheles* mosquitoes. Indeed, a variety of δ-endotoxins confers on *Bti* an insecticidal potential [45]. Moreover, cytolytic toxins present play an important role in the toxicity of *Bti* against dipteran larvae by increasing the toxicity and reducing the possibility of the appearance of resistance [46]. During the dry seasons a low density of *Anopheles* spp. was seen in both the LLIN + *Bti* and the LLIN only arms during the study period, likely due to changes in biotic and abiotic factors (sunlight, mud, dilution, crystals, rainfall, flushing), as observed in Burkina Faso, Côte d’Ivoire and Keya [27, 32, 33]. These seasonal variations are not likely to have affected negatively or biased the comparison of LLIN + *Bti* arm with LLIN arm as both study arms were subjected to the same environmental conditions across the seasons. Therefore, it could be advantageous to start larvicide applications during the dry season to reduce the exponential growth in mosquito numbers following the rains. In addition, the use of *Bti* during the dry season has the effect of reducing the amount of *Bti* required because the volume of water in the breeding sites is lower and the number of breeding sites during the dry season are fewer compared with the rainy season. Therefore, beginning *Bti* intervention during the dry season in the current study might have greatly improved its efficacy against *Anopheles* and *Culex* mosquito larvae.

The high impact of *Bti* applications in controlling *Anopheles* mosquito larvae observed in the present study significantly affected *An. gambiae* adult population biting rates, resulting in lower malaria transmission and clinical incidence in the LLIN + *Bti* compared to the LLIN-only arm. Indeed, before initiating the *Bti* intervention, in the villages where LLIN was previously the only vector control measure, the number of human-biting *An. gambiae* females and malaria cases were high. Once the *Bti* intervention was added to the use of LLINs, both EIR and the number of malaria cases dropped markedly in the LLIN + *Bti* treatment arm area. This significant reduction may relate to *Bti* applications which controlled *Anopheles* larvae, preventing them from reaching the adult stage and, thus, reducing *Plasmodium* transmission to local people. The parity rate of *An. gambiae* remained high in both study arms but was higher in the LLIN + *Bti* arm compared with LLIN-only arm, during the *Bti* applications. This may be explained by the lower larval density in the LLIN + *Bti* arm, reducing adult emergence and leading to a higher percentage of parous females of *An. gambiae* in this study arm compared to the LIIN-only arm. Indeed, the *Bti* lethal effect on the larvae, especially on the late instar, might have inhibited the emergence of *An. gambiae* female neonates in the LLIN + Bti arm, which continued to occur in the LLIN-only arm [47]. Parous females may also have possibly invaded the LLIN + *Bti* arm from neighbouring areas were *Bti* was not applied, as observed in Burkina Faso [47]. Conversely, the adult population of *An. gambiae* could be regenerated by the emergence of female neonate in LLIN-only arm, in which *Bti* was not applied. The lowest malaria prevalence in the *Bti*-treated villages was consistent with the low vector density and the comparatively low EIR. Additionally, EIR obtained in the current field trial showed that people have received fewer infective bites in the *Bti*-treated villages than in the LLIN-only villages. However, the annual EIR recorded in the *Bti*-treated villages was slightly high as an annual EIR above 1 ib/p/y could be enough to sustain *Plasmodium* transmission [48]. Nevertheless, the lower vector density and the strong decline in malaria incidence in the LLIN + *Bti* arm could be due to supplementary protection created locally by the *Bti* intervention. Indeed, this positive effect of *Bti* in protecting people against malaria was more pronounced in children under five who are more vulnerable to malaria infections.

Although LLIN + *Bti* showed overall high effectiveness against *An. gambiae* s.l*.* and malaria transmission in the present study, additional investigations are needed to address methodological limitations for better understanding the epidemiology of this disease in the target the villages. This study was conducted in villages with mean nets usage rates under 40%, only. Applying Bti in villages with low nets use rates (e.g., low and middle nets use rates) would likely help to strengthen them effectiveness and more beneficial for effective malaria control programs. *Plasmodium* transmission decreased significantly in the villages where *Bti* was applied with an EIR was still high (> 1 ib/p/y) in LLIN + Bti arm thus allowing possible residual malaria transmission in this area. Assessing the mosquito dispersal and possible invasion of villages within the LLIN + *Bti* trial arm with adult *An. gambiae* mosquitoes from neighbouring untreated breeding sites or areas may help to determine the buffer zone around treated villages to prevent recolonization [49]. This high rate of *An. gambiae* females parous in the LLIN + *Bti* could being justified emergence inhibition of neonate females. Moreover, assessment of *Plasmodium* infection among the whole populations through parasitological surveys in the villages in addition to the clinical cases among patients in the hospitals may allow to better understand the impact of the LLIN + *Bti* combination of malaria prevalence. Indeed, previous studies have reported that the number of *Plasmodium*-infected, asymptomatic carriers was high [27], or people suffering from malaria would not always go to health centre in villages. The use of *Bti* should be considered as an additional vector control intervention in combination with newer LLINs products (e.g., PermaNet 3.0, PermaNet Dual, Interceptor G2). Finally, large-scale trials of the combined use of *Bti* and LLINs to assess community acceptability and operational effectiveness against malaria vector control are needed to further validate this approach.

In summary, in the current community trial, the repeated application of *Bti* biolarvicide to *Anopheles* mosquito breeding sites in addition to LLINs in a highly endemic area was found to be highly effective in reducing *Plasmodium* transmission as well as malaria morbidity, Thus, integrated *Bti* and LLINs intervention could be seen as a promising approach to enhancing malaria vector control in Côte d’Ivoire [25]. Moreover, no adverse events and complains were reported among the sprayers and the inhabitants of villages treated with the *Bti* biolarvicide during and after the applications, and this might improve community acceptability and adherence for combination of *Bti* with LLINs. The use of water as solvent can reduce the environmental impact and costs of *Bti* treatment [50].

The use of malaria clinical health data collected from local health registers of a working document of health centres covering the study area could be a limitation of this study. A parasitological study in the target population in the study area could have generated more reliable data on malaria incidence. The absence of vector abundance data before the *Bti* intervention could be a limitation of this study, as this data could have allowed us to examine the effect of the *Bti* intervention on change in the relative abundance of different species of mosquitoes. The fact that this study was done in the context of low net use and pyrethroid only nets is a potential limitation as it is possible that with effective control of mosquitoes through high coverage with effective nets, the impact of larviciding would be much lower. Another study on Bti including a high coverage with effective nets could be confirmed the results of this study. It is also a limitation that only 4 villages were included. Lastly, a study on the cost of LSM intervention could be essential to better guide decision-makers.

## Conclusion

The current community study conducted in the savannah northern region of Côte d’Ivoire demonstrated that the repeated applications of the *Bti* biolarvicide to natural breeding sites of mosquitoes, in addition to the use of LLINs in households, resulted in an effective control of *Anopheles* larvae. This led to a significant reduction in *An. gambiae* density and malaria transmission and clinical incidence among the target population in the LLIN + *Bti* arm compared to in the LLIN-only arm. These outcomes suggested that, while the use of LLINs against vectors is indispensable as a malaria control intervention, it may require other complementary vector control tools, including larviciding, to maximize effectiveness, especially where LLIN use rates are low and where some of the malaria vectors may bite early or outdoors. Therefore, integrating a *Bti-*based LSM programme should be considered as a complementary measure to LLIN mass distribution to help drive malaria elimination efforts in Côte d'Ivoire and other malaria endemic settings in sub-Saharan Africa. In addition, *Bti* applications in the presence of LLINs had a positive effect in reducing both *Anopheles* and *Culex* mosquitoes, thus reducing malaria transmission and mosquito nuisance biting, and this may be beneficial to the optimal coverage of LLINs, as reduction of mosquito nuisance biting is key to the acceptance and adoption of vector tools. Ultimately, *Bti* biolarvicide applications in association with the distribution if LLINs appears to be a promising tool targeting the major vector *An. gambiae* larvae for the control and the elimination of malaria, even in areas with low use or coverage of LLINs.

### Supplementary Information

**Additional file 1: Fig. S1.** Variation in the average density of larvae of *Anopheles* spp*.* in the study arms, in Napié area in northern Côte d’Ivoire from March 2019 to February 2020. LLIN: long-lasting insecticidal nets; *Bti*: *Bacillus thuringiensis israelensis;* Trt: treatment.

**Additional file 2: Fig. S2.** Variation in the average density of larvae of *Culex* spp*.* in the study arms, in Napié area in northern Côte from March 2019 to February 2020. LLIN: long-lasting insecticidal nets; *Bti*: *Bacillus thuringiensis israelensis;* Trt: treatment.

**Additional file 3: Fig. S3.** Variation parity rate in *An. gambiae *s.l. in the study arms, in northern Côte d’Ivoire. LLIN: long-lasting insecticide-treated nets; *Bti*: *Bacillus thuringiensis israelensis;* Trt: treatment.

**Additional file 4: Table S1.** Variation in some transmission parameters in the study area, in Napié area in northern Côte d’Ivoire from March 2019 to February 2020. LLIN: long-lasting insecticide-treated nets; *Bti*: *Bacillus thuringiensis israelensis;* Trt: treatment.

**Additional file 5: Table S2.** Distribution of the rate of use of nets according to the different age groups from March 2019 to February 2020, in Napié area in northern Côte d’Ivoire. Long-lasting insecticidal nets; *Bti*: *Bacillus thuringiensis israelensis;* n: number; %: percentage; RR: rate ratio.

## Data Availability

Open access.
