# Supplementary figures and images for: Combined use of long-lasting insecticidal nets and Bacillus thuringiensis israelensis larviciding, a promising integrated approach against malaria transmission in northern Côte d'Ivoire
Source: Malar J. 2024 May 29;23:168. doi: 10.1186/s12936-024-04953-8 (PMC11137964; doi:10.1186/s12936-024-04953-8)

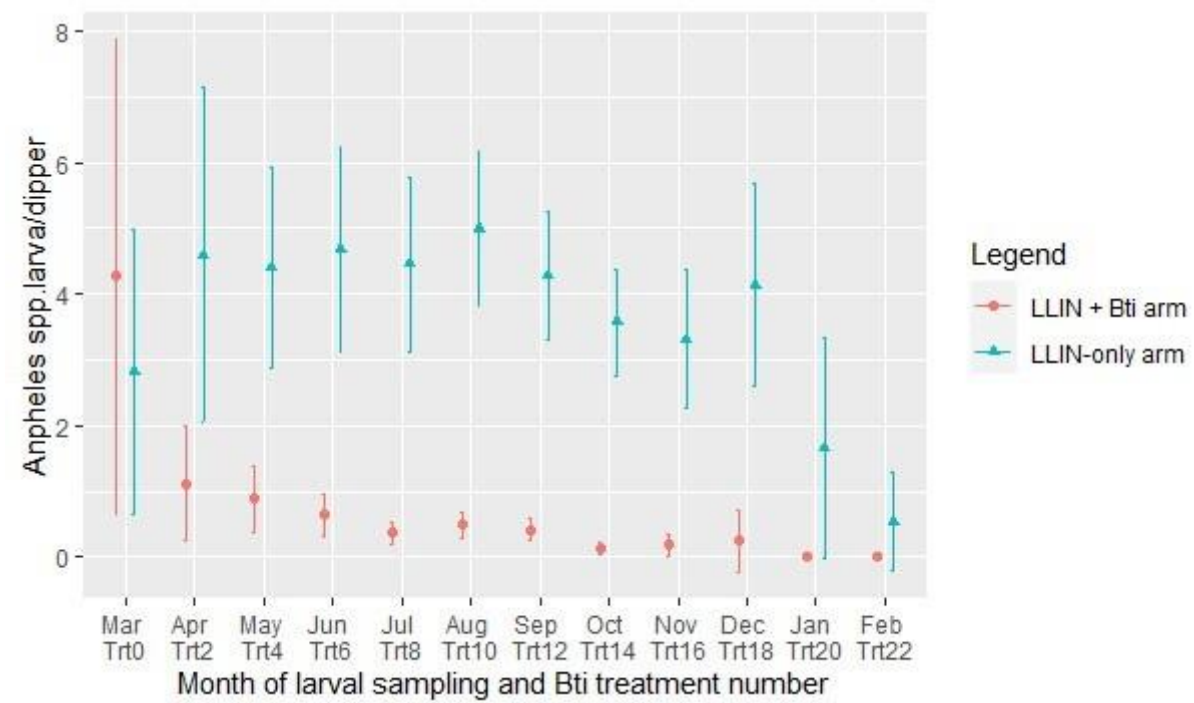

Supplement: Supplementary file 1 — Additional file 1: Fig. S1. Variation in the average density of larvae of Anopheles spp. in the study arms, in Napié area in northern Côte d’Ivoire from March 2019 to February 2020. LLIN: long-lasting insecticidal nets; Bti: Bacillus thuringiensis israelensis; Trt: treatment. [file 12936_2024_4953_MOESM1_ESM.pdf]

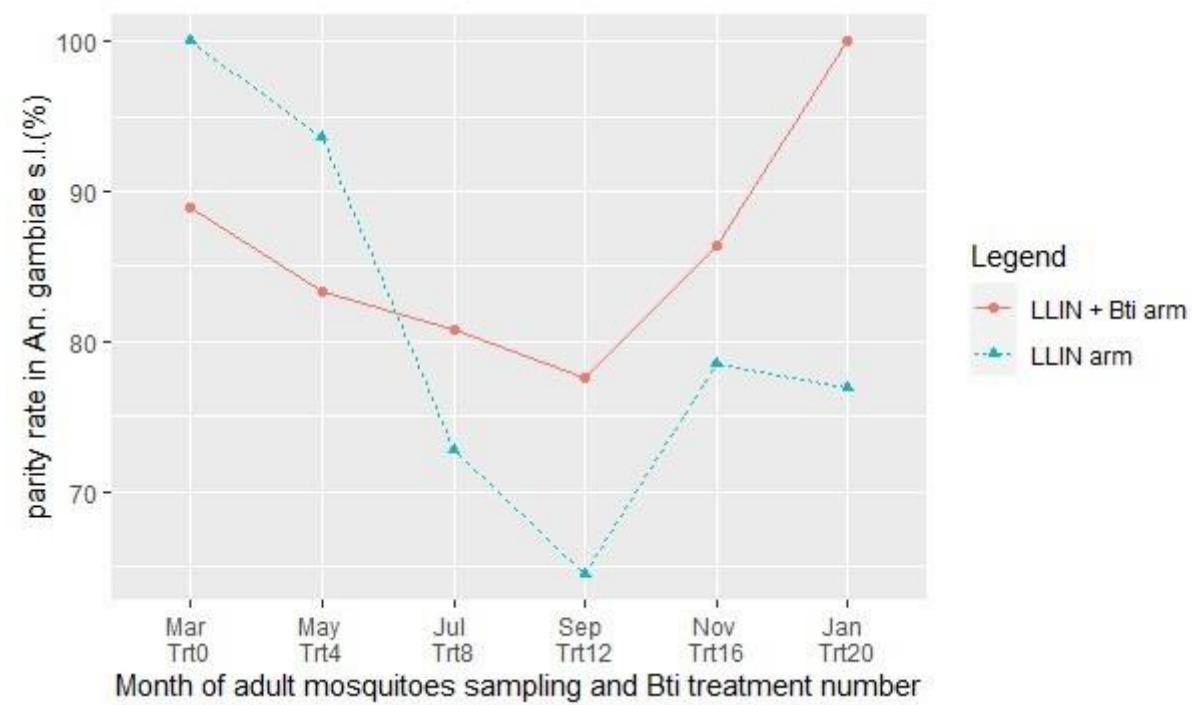

Supplement: Supplementary file 3 — Additional file 3: Fig. S3. Variation parity rate in An. gambiae s.l. in the study arms, in northern Côte d’Ivoire. LLIN: long-lasting insecticide-treated nets; Bti: Bacillus thuringiensis israelensis; Trt: treatment. [file 12936_2024_4953_MOESM3_ESM.pdf]
