# Supplementary material for: Combined use of long-lasting insecticidal nets and Bacillus thuringiensis israelensis larviciding, a promising integrated approach against malaria transmission in northern Côte d'Ivoire
Source: Malar J. 2024 May 29;23:168. doi: 10.1186/s12936-024-04953-8 (PMC11137964; doi:10.1186/s12936-024-04953-8)
Supplement: Supplementary file 2 — Additional file 2: Fig. S2. Variation in the average density of larvae of Culex spp. in the study arms, in Napié area in northern Côte from March 2019 to February 2020. LLIN: long-lasting insecticidal nets; Bti: Bacillus thuringiensis israelensis; Trt: treatment. [file 12936_2024_4953_MOESM2_ESM.pdf]

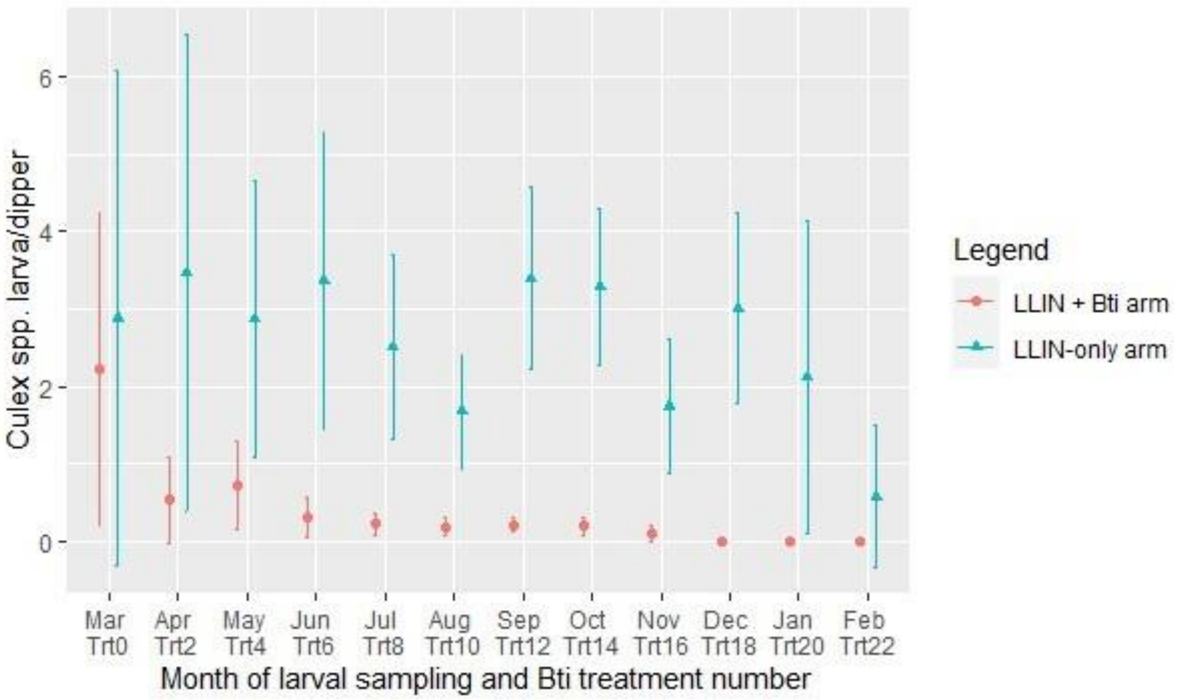

Additional file 2 Fig.2: Variation in the average density of larvae of *Culex* spp. in the study arms, in Napié area in northern Côte d'Ivoire from March 2019 to February 2020. LLIN: long-lasting insecticidal nets; *Bti*: *Bacillus thuringiensis israelensis;* Trt: treatment
