# Supplementary material for: Combined use of long-lasting insecticidal nets and Bacillus thuringiensis israelensis larviciding, a promising integrated approach against malaria transmission in northern Côte d'Ivoire
Source: Malar J. 2024 May 29;23:168. doi: 10.1186/s12936-024-04953-8 (PMC11137964; doi:10.1186/s12936-024-04953-8)
Supplement: Supplementary file 4 — Additional file 4: Table S1. Variation in some transmission parameters in the study area, in Napié area in northern Côte d’Ivoire from March 2019 to February 2020. LLIN: long-lasting insecticide-treated nets; Bti: Bacillus thuringiensis israelensis; Trt: treatment. [file 12936_2024_4953_MOESM4_ESM.pdf]

Additional file 4 Table S1: Variations in some transmission parameters in the study area from March 2019 to February 2020

<table>
<tr><td colspan="2" rowspan="3">An. gambiae s.l.</td><td>LLIN +Bti</td><td>LLIN</td><td>Total</td></tr>
<tr><td>n (%)</td><td>n (%)</td><td>n (%)</td></tr>
<tr><td>375 (14.5)</td><td>2,212 (85.5)</td><td>2,587(100)</td></tr>
<tr><td rowspan="5">Origin bloodfed</td><td>Human</td><td>100 (25.7)</td><td>213 (54.8)</td><td>313 (80.5)</td></tr>
<tr><td>Livestock (bovine, sheep, and goat)</td><td>8 (2.1)</td><td>12 (3.1)</td><td>20 (5.1)</td></tr>
<tr><td>Mixed blood (Human and livestock)</td><td>6 (1.5)</td><td>18 (4.6)</td><td>24 (6.2)</td></tr>
<tr><td>Bloodfed origin no known</td><td>14 (3.6)</td><td>18 (4.6)</td><td>32 (8.2)</td></tr>
<tr><td>n</td><td>128 (32.9)</td><td>10.44</td><td>389 (100)</td></tr>
<tr><td rowspan="3">An. gambiae s.l.</td><td>An. gambiae s.s.</td><td>89 (28.9)</td><td>204 (66.2)</td><td>293 (95.1)</td></tr>
<tr><td>An. coluzzii</td><td>11(3.6)</td><td>4 (1.3)</td><td>15 (4.9)</td></tr>
<tr><td>n</td><td>93 (30.2)</td><td>215 (68.8)</td><td>308 (100)</td></tr>
<tr><td rowspan="3">P. falciparum infection</td><td>An. gambiae s.s.</td><td>9 (2.9)</td><td>24 (7.8)</td><td>33 (10.7)</td></tr>
<tr><td>An. coluzzii</td><td>0</td><td>2 (0.6)</td><td>2 (0.6)</td></tr>
<tr><td>n</td><td>9 (2.9)</td><td>26 (8.4)</td><td>35 (11.4)</td></tr>
<tr><td colspan="2">Sleeper</td><td>747(48.9)</td><td>779 (51.1)</td><td>1526 (100)</td></tr>
<tr><td colspan="2">Entomological infection rate EIR (ib/p/n)</td><td>0.004</td><td>0.13</td><td>0.15</td></tr>
<tr><td colspan="2">Entomological infection rate EIR (ib/p/y)</td><td>1.36</td><td>47.70</td><td>56.78</td></tr>
</table>

LLIN: long-lasting insecticidal nets; *Bti*: *Bacillus thuringiensis var. israelensis; n:number; %:percentage*

EIR: Entomological infection rate; ib/p/n: infection bite per person per night
