# Supplementary material for: Combined use of long-lasting insecticidal nets and Bacillus thuringiensis israelensis larviciding, a promising integrated approach against malaria transmission in northern Côte d'Ivoire
Source: Malar J. 2024 May 29;23:168. doi: 10.1186/s12936-024-04953-8 (PMC11137964; doi:10.1186/s12936-024-04953-8)
Supplement: Supplementary file 5 — Additional file 5: Table S2. Distribution of the rate of use of nets according to the different age groups from March 2019 to February 2020, in Napié area in northern Côte d’Ivoire. Long-lasting insecticidal nets; Bti: Bacillus thuringiensis israelensis; n: number; %: percentage; RR: rate ratio. [file 12936_2024_4953_MOESM5_ESM.pdf]

Additional file 5 Table S2 : Distribution of nets use rate according to the different age groups from March 2019 to February 2020, in Napié area in northern Côte d'Ivoire

		LLIN + *Bti*	LLIN-only		Rateratio test	
Age groups	Use net	n (%)	n (%)	Total (%)	RR	P-value
< 5 years	Yes	186 (41.2)	195 (43.1)	381 (84.3)	0.95	0.682
	No	38 (8.4)	33 (7.3)	71 (15.7)		
	n	224 (49.6)	228 (50.4)	452 (100)		
5-15 years	Yes	250 (36.3)	254 (36.9)	504 (73.1)	1.02	0.8937
	No	121 (17.6)	64 (9.3)	185 (26.9)		
	n	371 (53.8)	318 (46.2)	689 (100)		
> 15 years	Yes	554 (42.8)	433 (33.5)	987 (76.3)	1.28	< 0.001
	No	237 (18.3)	70 (5.4)	307 (23.7)		
	n	791 (61.1)	503 (38.9)	1,294 (100)		
All ages	Yes	990 (40.7)	882 (36.2)	1,872 (76.9)	1.12	0.013
	No	396 (16.3)	167 (6.9)	563 (23.1)		
	n	1,386 (56.9)	1,049 (43.1)	2,435 (100)		

n: Number; %: Percentage; LLIN: long-lasting insecticidal-treated net; *Bti*: *Bacillus thuringiensis var. israelensis.* RR: rate ratio
